# Risk of heart failure in patients with schizophrenia or bipolar disorder: A systematic review and meta-analysis

**DOI:** 10.12669/pjms.42.8.16392

**Published:** 2026-08

**Authors:** Qifeng Dong, Xing Ye

**Affiliations:** 1 Qifeng Dong, Department of General Male Psychiatry, Jiaxing Kangci Hospital, Jiaxing, Zhejiang Province 314500, P.R. China; 2 Xing Ye, Department of General Male Psychiatry, Jiaxing Kangci Hospital, Jiaxing, Zhejiang Province 314500, P.R. China

**Keywords:** Cardiac, Cardiovascular, Risk, Severe mental illness

## Abstract

**Background and Objective:**

Individuals with schizophrenia and bipolar disorder experience excess cardiovascular morbidity and premature mortality; however, the specific risk of incident heart failure has not been comprehensively quantified. We conducted a systematic review and meta-analysis to evaluate the risk of heart failure in these populations.

**Methodology:**

A search was conducted on PubMed, Embase, Scopus, and Web of Science databases from inception through February 10, 2026, with no language restrictions, for cohort studies that reported adjusted estimates of the risk of incident heart failure in patients with schizophrenia or bipolar disorder. Random-effects meta-analyses were performed to obtain risk ratio (RR). Heterogeneity was assessed using the I² statistic.

**Results:**

Seven retrospective cohort studies met the inclusion criteria. Schizophrenia was associated with a significantly increased risk of incident heart failure (RR: 1.50, 95% CI 1.35–1.67; I² = 74.9%). Bipolar disorder was associated with an even greater risk (RR: 1.95, 95% CI 1.31–2.91; I² = 80.1%). Sensitivity analyses confirmed the robustness of pooled estimates.

**Conclusions:**

Both schizophrenia and bipolar disorder are independently associated with substantially elevated risks of incident heart failure. High heterogeneity in the meta-analysis warrants caution in interpreting the results.

**Registration No:** PROSPERO (No. CRD420261303975).

## INTRODUCTION

Cardiovascular disease (CVD) continues to be the predominant cause of mortality globally, responsible for nearly one-third of all deaths despite advancements in preventive measures and therapeutic interventions.^1^ Although there has been a decline in overall CVD mortality within many high-income nations, this decline has not been equitably experienced across different population groups. Patients with severe mental illnesses, notably schizophrenia and bipolar disorder, have shown disproportionately elevated rates of cardiovascular morbidity and premature death.^2^,^3^ In cases of schizophrenia, CVD is the primary cause of natural death, significantly contributing to a reduction in life expectancy ranging from 13 to 25 years.^4^ Similarly, bipolar disorder has been linked to increased cardiovascular morbidity and early mortality, with increased risk of myocardial infarction, stroke and other cardiac diseases.^4^,^5^

A number of mechanisms have been proposed to explain the elevated cardiovascular burden in these diseases. Patients diagnosed with schizophrenia and bipolar disorder show a high prevalence of modifiable cardiovascular risk factors, including obesity, hypertension, dyslipidemia, diabetes mellitus, physical inactivity, and smoking.^6^ Moreover, psychotropic medications, particularly antipsychotics and mood stabilisers, are associated with adverse metabolic effects that may further increase cardiovascular risk.^7^ Importantly, heart failure has emerged as a significant clinical outcome in both conditions. Data from a nationwide Korean cohort indicated that bipolar disorder was linked with two times higher risk of hospitalisation due to heart failure,^8^ while German primary care data revealed a markedly elevated 10-year incidence of heart failure among individuals with schizophrenia.^4^ Despite such studies, there is no comprehensive up-to-date evidence on the actual risk of heart failure in these diseases. Many prior investigations have primarily focused on composite cardiovascular outcomes or mortality, with comparatively less emphasis on incident heart failure as an independent endpoint.^9^-^11^

Furthermore, previous meta-analyses examining the risk of heart failure in schizophrenia and bipolar disorder have been limited by either a small number of included studies or by combining cross-sectional and cohort studies in a single analysis.^12^,^13^ Given that temporality can be accurately assessed only through cohort studies, integrating different study designs can lead to biased results. Such evidence is necessary to clarify disorder-specific associations and to provide a more precise estimate of heart failure risk in these high-risk psychiatric populations. It is especially needed for clinicians and nursing personnel play a pivotal role in bridging the gap between psychiatric and cardiovascular care in patients with severe mental illness. As frontline healthcare providers, nurses are uniquely positioned to perform routine cardiometabolic screening, monitor medication-related adverse effects, reinforce lifestyle modification strategies, and promote adherence to both psychiatric and cardiovascular therapies. Consequently, this systematic review and meta-analysis aimed to evaluate the pooled risk of incident heart failure in patients with schizophrenia and bipolar disorder, utilising adjusted estimates derived from cohort studies.

## METHODOLOGY

The review process is reported based on PRISMA (Preferred Reporting Items for Systematic Reviews and Meta-Analyses) guidelines.^14^ Protocol was registered on PROSPERO (No. CRD420261303975).

### Eligibility criteria:

Studies were eligible if they were based on the following criteria.

- Prospective or retrospective cohort design.
- Inclusion of adults diagnosed with schizophrenia or bipolar disorder.
- Presence of a comparison group without the respective psychiatric disorder.
- Assessment of incident heart failure as an outcome.
- Reporting of adjusted effect estimates (hazard ratios (HR), risk ratios (RR), or incidence rate ratios) with corresponding 95% confidence intervals (CI).

### Exclusion criteria:

They were excluded if they evaluated only prevalent cardiovascular disease, used cross-sectional or case–control designs, included exclusively patients with pre-existing heart failure at baseline, or failed to report adjusted estimates. When studies reported schizophrenia and bipolar disorder separately, each exposure group was treated as an independent dataset for quantitative synthesis.

### Search strategy:

Two independent reviewers conducted a literature search across PubMed, Embase, Scopus, and Web of Science from their inception until February 10, 2026, without any language restrictions. The specific search strategies are shown in Supplementary Table-I. We also examined Google Scholar and the reference lists of included studies for additional relevant articles. All references were organized and deduplicated using EndNote (version 21). The screening process involved two reviewers independently evaluating all search results in three steps:

**Supplementary Table-I:** Search Strategy.

***PubMed Search Strategy***
((“Schizophrenia”[Mesh] OR schizophrenia[Title/Abstract] OR “schizoaffective disorder”[Title/Abstract])
OR
(“Bipolar Disorder”[Mesh] OR “bipolar disorder”[Title/Abstract] OR “manic-depressive”[Title/Abstract]))
AND
(“Heart Failure”[Mesh] OR “heart failure”[Title/Abstract] OR “cardiac failure”[Title/Abstract]
OR “congestive heart failure”[Title/Abstract] OR HF[Title/Abstract])
AND
(“Cohort Studies”[Mesh] OR cohort[Title/Abstract] OR longitudinal[Title/Abstract]
OR “follow-up”[Title/Abstract] OR incidence[Title/Abstract])
***Embase Search Strategy***
(‘schizophrenia’/exp OR schizophrenia:ti,ab OR ‘schizoaffective disorder’:ti,ab)
OR
(‘bipolar disorder’/exp OR ‘bipolar disorder’:ti,ab OR ‘manic depressive’:ti,ab)
AND
(‘heart failure’/exp OR ‘heart failure’:ti,ab OR ‘cardiac failure’:ti,ab
OR ‘congestive heart failure’:ti,ab)
AND
(‘cohort analysis’/exp OR cohort:ti,ab OR longitudinal:ti, ab OR ‘follow up’:ti,ab OR incidence:ti,ab)
***Scopus Search Strategy***
(TITLE-ABS-KEY(schizophrenia OR “schizoaffective disorder”
OR “bipolar disorder” OR “manic depressive”))
AND
(TITLE-ABS-KEY(“heart failure” OR “cardiac failure”
OR “congestive heart failure” OR HF))
AND
(TITLE-ABS-KEY(cohort OR longitudinal OR “follow up” OR incidence))
***Web of Science Search Strategy***
TS=(schizophrenia OR “schizoaffective disorder”
OR “bipolar disorder” OR “manic depressive”)
AND
TS=(“heart failure” OR “cardiac failure”
OR “congestive heart failure” OR HF)
AND
TS=(cohort OR longitudinal OR “follow up” OR incidence)

- Removing duplicates,
- Screening titles and abstracts,
- Reviewing full texts for eligibility. Any disagreements were resolved through discussion.

### Data extraction:

A standardized data extraction form was used by two reviewers to independently perform data extraction. Extracted variables included study identifier, country, study design, psychiatric disorder assessed, sample sizes of exposed and control groups, matching variables (if applicable), age distribution, diagnostic criteria for psychiatric disorders, heart failure definition and coding algorithms, follow-up duration, covariates included in multivariable models, and adjusted effect estimates with 95% CI. When multiple models were presented, the most fully adjusted estimate was selected to minimize residual confounding.

### Risk of bias assessment:

The reviewers assessed risk of bias using the Newcastle–Ottawa Scale (NOS). This tool assesses three key areas: how study groups are selected, the comparability between cohorts, and the methods used to assess outcomes. Each study could earn up to nine stars. Studies with seven to nine stars were classified as high quality, those with five to six stars as moderate quality, and studies with fewer than five stars as low quality.

### Statistical analysis:

Separate meta-analyses were conducted for schizophrenia and bipolar disorder. The included studies reported data as HR, RR, or incidence rate ratio, and because heart failure incidence was relatively low across populations, these effect measures were considered comparable and pooled as RR with 95% CI. Effect sizes were logarithmically transformed prior to pooling. Standard errors were calculated from reported confidence intervals using conventional methods. Pooled estimates were generated using a random-effects model based on the DerSimonian and Laird approach, which accounts for both within-study and between-study variability. Statistical heterogeneity was assessed using the I² statistic. I² values were interpreted as representing low (<25%), moderate (25–50%), or substantial (>50%) heterogeneity. Robustness of pooled estimates was evaluated using leave-one-out sensitivity analyses. Publication bias was not assessed due to small number of studies. R software was used for the analysis.

## RESULTS

**Fig.1:**
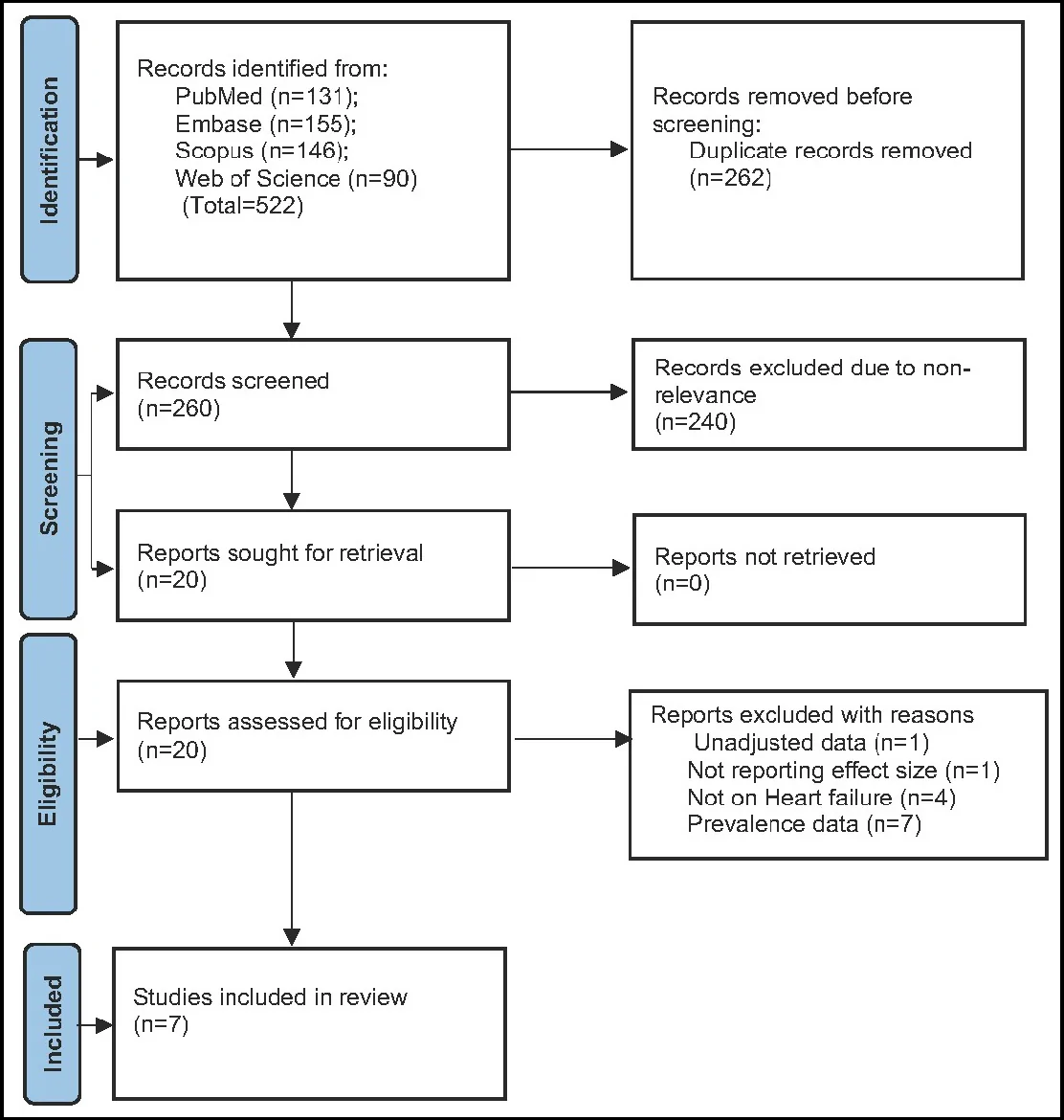
Study flowchart.

The process of selecting studies is shown in Fig.1. After removing duplicates and screening titles and abstracts, 20 full-text articles were evaluated for eligibility. Thirteen studies were excluded mainly because they had a cross-sectional design, focused on prevalent instead of incident heart failure, or lacked adjusted effect estimates. In the end, seven studies fulfilled the inclusion criteria.^3^,^4^,^8^,^15^-^18^ The agreement between reviewers for selecting studies was very high (kappa=0.95).

Baseline details of studies are shown in Table-I. All included studies employed retrospective cohort designs and were conducted in North America (Canada, United States), Europe (Germany), and East Asia (Taiwan, South Korea, Japan). Sample sizes varied substantially, ranging from 146 to over four million participants in control cohorts. Follow-up duration ranged from three to 11.2 years. Psychiatric diagnoses were primarily ascertained using ICD coding algorithms within administrative or national health insurance databases, with some studies incorporating physician documentation or medication records. Incident heart failure was identified using validated ICD-based definitions, and all studies reported multivariable-adjusted risk estimates accounting for key demographic and cardiovascular confounders.

**Table-I:** Baseline details of included studies.

Study ID, Country	Study Design	Disease Studied	Sample Size (Exposure)	Sample Size (Control)	Matching Variables	Age (Exposure) (yrs)	Age (Control) (yrs)	Diagnosis Method (Psychiatric Disorder)	Diagnosis Method (Heart Failure)	Adjusted Effect Size	Follow-up Duration (yrs)	Variables Adjusted
Curkendall 2004 Canada	RC	Schizo-phrenia	3022	12088	Age, sex	Median 47	NR (mat-ched)	ICD codes	ICD codes	RR 1.6 (95% CI 1.2–2.0)	3	Age, sex, CVD history, HTN, hyperlipidemia, diabetes, pulmonary disease
McDermott 2005 USA	RC	Schizo-phrenia	357	2083	Age	Mean 45.6	Mean 43.5	ICD + physician documentation	Medical record review	RR 2.27 (95% CI 1.49–3.45)	7.9	Age, race, gender, smoking, comorbidities
McDermott 2005 USA	RC	Bipolar disorder	146	2083	Age strata	Mean 43.8	Mean 43.5	ICD + physician documentation	Medical record review	RR 1.73 (95% CI 0.88–3.39)	7.5	Age, race, gender, smoking, comorbidities
Chen 2022 Taiwan	RC	Bipolar disorder	11884	47536	Age, sex	13–40	NR (mat-ched)	ICD codes	ICD codes	IRR 2.62 (95% CI 1.50–4.57) – 5th year	5	Age, sex, urbanization, employment, CCI
Yoo 2023 South Korea	RC	Schizo-phrenia	10312	2447 386	None	NR	NR	ICD codes	ICD codes	HR 1.405 (95% CI 1.302–1.516)	7.09	Demographics, lifestyle, comorbidities, DM variables
Yoo 2023 South Korea	RC	Bipolar disorder	7284	2447 386	None	NR	NR	ICD codes	ICD codes	HR 1.406 (95% CI 1.301–1.520)	7.09	Demographics, lifestyle, comorbidities, DM variables
Komuro 2024 Japan	RC	Schizo-phrenia	31341	409 3167	None	Median 44	Median 44	ICD codes	ICD codes	HR 1.60 (95% CI 1.50–1.70)	3.5	Age, BMI, HTN, diabetes, dyslipidemia, lifestyle
Lee 2024 South Korea	RC	Bipolar disorder	11329	11329	Age, sex	Mean 34.06	Mean 34.06	ICD + medication	ICD codes	HR 2.526 (95% CI 1.788–3.567)	11.2	CCI, income, disability
Rodemer 2025 Germany	RC	Schizo-phrenia	12527	62635	Propensity score (age, sex, index year, comorbidities)	Mean 49.8	Mean 50.0	ICD codes	ICD codes	HR 1.33 (95% CI 1.20–1.48)	Up to 10	Propensity score matching variables

RC, retrospective cohort; BMI, body mass index; HTN, hypertension; CCI, Charlson comorbidity index; HTN, hypertension; ICD< international classification of diseases; yrs, years.

### Schizophrenia:

**Fig.2:**
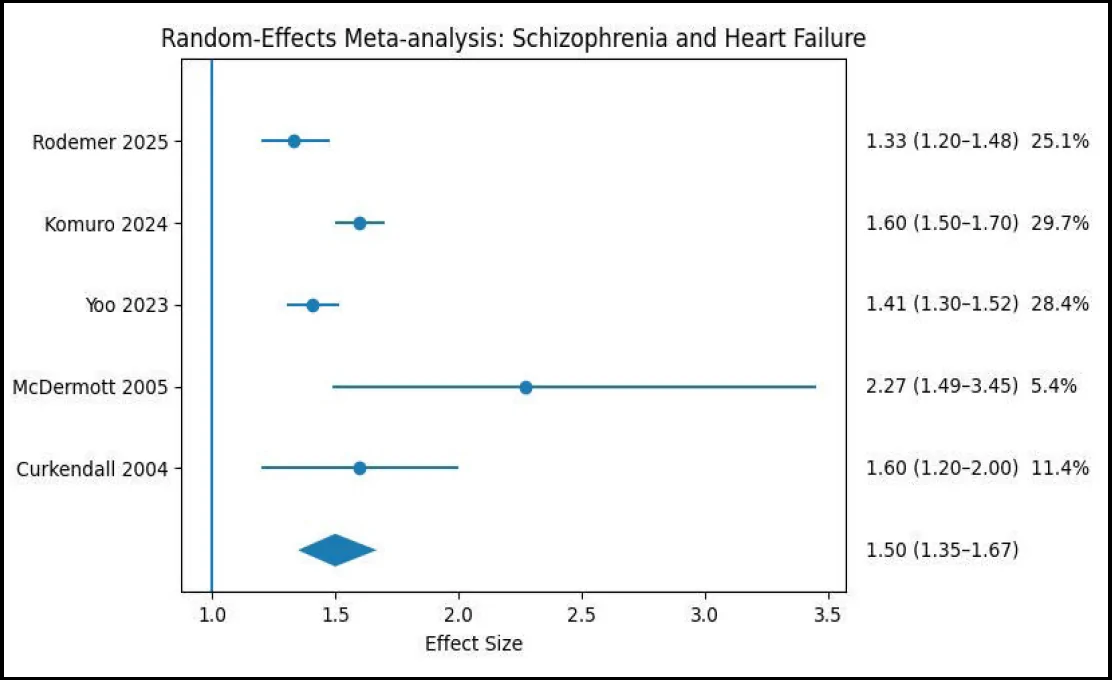
Meta-analysis of the risk of heart failure in schizophrenia.

Five studies contributed to the schizophrenia meta-analysis. On random-effects modelling, schizophrenia was associated with a significantly increased risk of incident heart failure, with a pooled RR of 1.50 (95% CI 1.35–1.67) (Fig.2). The meta-analysis had substantial heterogeneity (I² = 74.9%). Leave-one-out sensitivity analysis demonstrated that exclusion of any single study did not alter the pooled estimate, with resultant pooled effect sizes remaining 1.49 to 1.56. Although heterogeneity was attenuated in some iterations, the association between schizophrenia and heart failure remained robust, indicating that no individual study disproportionately influenced the overall result (Supplementary Table-II).

**Supplementary Table-II:** Sensitivity Analysis- Schizophrenia.

Study Excluded	Resultant Effect Size (95% CI)	I²
Curkendall 2004	1.490 (1.324–1.678)	80.8%
McDermott 2005	1.465 (1.326–1.618)	75.1%
Yoo 2023	1.555 (1.338–1.807)	75.6%
Komuro 2024	1.451 (1.290–1.632)	57.5%
Rodemer 2025	1.559 (1.385–1.755)	70.9%

### Bipolar Disorder:

**Fig.3:**
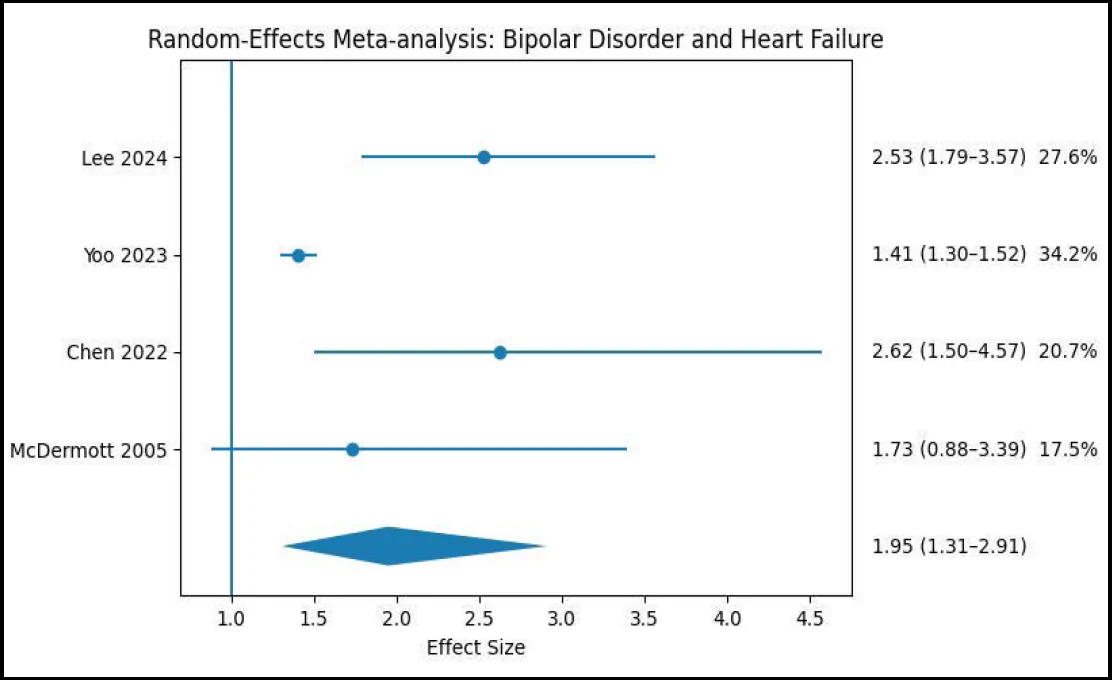
Meta-analysis of the risk of heart failure in bipolar disorder.

Four studies contributed to the bipolar disorder meta-analysis. Random-effects modelling demonstrated that bipolar disorder was associated with a significantly increased risk of incident heart failure, with a pooled RR of 1.95 (95% CI 1.31–2.91) (Fig.3). The meta-analysis had substantial heterogeneity (I² = 80.1%). Leave-one-out sensitivity analysis demonstrated that the pooled estimate remained statistically significant after excluding each study in turn, with resulting pooled estimates ranging from 1.7 to 2.4 (Supplementary Table-III).

**Supplementary Table-III:** Sensitivity Analysis– Bipolar Disorder.

Study Excluded	Resultant Effect Size (95% CI)	I²
McDermott 2005	2.017 (1.242–3.278)	86.5%
Chen 2022	1.802 (1.161–2.798)	81.5%
Yoo 2023	2.399 (1.833–3.139)	0.0%
Lee 2024	1.726 (1.165–2.559)	60.2%

### Risk of bias analysis:

Overall methodological quality was high (Supplementary Table-IV). Most studies achieved the maximum score in the selection domain, reflecting representative population-based cohorts and appropriate ascertainment of psychiatric exposure. All studies demonstrated adequate comparability, with adjustment for key confounders. Outcome assessment was generally robust, with heart failure identified using validated administrative coding algorithms and sufficient follow-up duration to permit incident case detection. Total NOS scores ranged from 7-9.

**Supplementary Table-IV:** Risk of Bias Assessment.

Study	Selection (4)	Comparability (2)	Outcome (3)	Total (9)
Curkendall 2004	4	2	3	9
McDermott 2005	3	2	2	7
Chen 2022	4	2	3	9
Yoo 2023	4	2	3	9
Komuro 2024	4	2	3	9
Lee 2024	4	2	3	9
Rodemer 2025	4	2	3	9

## DISCUSSION

This systematic review and meta-analysis of cohort studies found that both schizophrenia and bipolar disorder are independently linked to a significantly higher risk of developing heart failure. Specifically, patients with schizophrenia have a 50% increased risk, while those with bipolar disorder face nearly double the risk compared to individuals without these psychiatric conditions. The consistent findings across all studies, along with sensitivity analyses, confirm the robustness of these results.

Consistent with our results, several previous meta-analyses have also shown that individuals with severe mental illness have a higher risk of cardiovascular diseases. A large-scale meta-analysis including over three million patients with severe mental illness reported a significantly higher incidence of cardiovascular disease compared to controls, with a pooled HR indicating an elevated risk of coronary heart disease, cerebrovascular disease, and congestive heart failure (HR: 2.10, 95% CI 1.64–2.70) among individuals with schizophrenia or bipolar disorder.^13^

Nonetheless, this study was limited by the availability of data pertaining to the relationship between heart failure and schizophrenia or bipolar disorder. Other comprehensive reviews have highlighted the considerable burden of cardiovascular morbidity and mortality associated with serious mental illness, noting approximately a twofold increase in cardiovascular mortality relative to the general population, as well as a higher incidence of various cardiovascular outcomes, including myocardial infarction and stroke.^9^ An earlier meta-analysis focusing specifically on schizophrenia identified an elevated pooled risk for incident congestive heart failure (RR = 1.81, 95% CI 1.42–2.29), along with increased risks for stroke and overall cardiovascular disease.^12^ Despite the inclusion criteria restricting the analysis to cohort studies, this meta-analysis erroneously included both cross-sectional and cohort studies, thereby compromising the validity of its conclusions. The present study provides stronger evidence than prior reviews, as it focused solely on cohort studies and conducted an updated literature search to include recent studies and provide separate evidence for schizophrenia and bipolar disorder.

Substantial heterogeneity between studies was identified in both analyses, with I² values of 74.9% for schizophrenia and 80.1% for bipolar disorder. This heterogeneity is likely attributable to multiple factors. Variations in population characteristics, including age distribution, baseline cardiovascular risk, and comorbidity burden, may partially account for differences in effect estimates. Additionally, disparities in follow-up duration and outcome definitions, ranging from claims-based diagnostic coding to hospitalisation-defined heart failure, may have influenced the results. Other contributing factors include differences in the severity and chronicity of psychiatric illness, which were not consistently reported, as well as variability in exposure to psychotropic medications such as second-generation antipsychotics, mood stabilisers, and polypharmacy, all of which possess distinct cardiometabolic risk profiles.

Recent meta-analyses have shown that use of antipsychotic drugs is associated with increased risk of cardio-cerebrovascular mortality,^19^ while there have been mixed results on the risk of cardiac diseases like myocardial infarction.^20^,^21^ Moreover, residual confounding related to socioeconomic status, healthcare accessibility, lifestyle behaviours (e.g., smoking, diet, physical inactivity), and adherence to cardiovascular prevention strategies may not have been uniformly measured or controlled across studies. Surveillance bias is also conceivable, given that patients with severe mental illness may experience increased healthcare contact, leading to earlier detection of conditions, or, alternatively, underdiagnosis of somatic illnesses. Despite this variability, the overall direction of the effect size was similar across studies, and sensitivity analyses supported the robustness of the pooled estimates.

Multiple mechanisms have been linked to the higher risk of heart failure in both conditions. Chronic low-grade inflammation, common to both diseases, involves elevated cytokines like IL-6, TNF-α, and IL-8, which promote myocardial fibrosis, left ventricular hypertrophy, and endothelial dysfunction.^22^,^23^ In schizophrenia, this inflammation leads to concentric cardiac remodelling, characterized by reduced biventricular volumes, increased septal thickness, and higher left ventricular concentricity. These promote collagen deposition and impair cardiac function, thereby increasing the risk of heart failure.^24^ Similarly, in bipolar disorder, factors such as oxidative stress, mitochondrial dysfunction, hypothalamic-pituitary-adrenal axis dysregulation, and genetic factors contribute to atherogenesis, arterial stiffness, and structural changes, including increased left ventricular mass index, ultimately leading to systolic and diastolic dysfunction.^23^,^25^

Additionally, autonomic nervous system dysregulation, frequently observed in severe mental illness, results in sympathetic overactivation and diminished heart rate variability, which promote hypertension, arrhythmias, and progressive myocardial stress.^26^ Emerging evidence suggests shared genetic factors between schizophrenia and cardiovascular disease, involving overlapping pathways implicated in heart failure and sigma-1 receptor–related mechanisms.^27^ Furthermore, metabolic disturbances play a significant role; individuals with schizophrenia and bipolar disorder exhibit higher incidences of obesity, insulin resistance, Type-2 diabetes, and dyslipidaemia, conditions directly contributing to coronary artery disease and diabetic cardiomyopathy.^28^ Lastly, psychotropic medications, particularly second-generation antipsychotics and certain mood stabilisers, can exacerbate weight gain, glucose dysregulation, and lipid abnormalities, thereby amplifying cardiovascular risk over time.^7^ Overall, several of these mechanisms may work in synchrony to increase the risk of heart failure in both diseases.

### Limitations:

First, all the studies included were observational, and even after adjusting for multiple variables, some confounding factors, especially related to lifestyle, illness severity, and use of psychotropic medications, may still influence the outcomes. Second, diagnoses of heart failure and psychiatric conditions were mainly based on administrative coding algorithms, which could lead to misclassification. Third, there was notable heterogeneity across studies, likely due to differences in population characteristics, healthcare systems, and outcome definitions. Fourth, data on medication doses, the length of psychiatric illness, and detailed cardiac phenotypes (such as systolic vs. diastolic heart failure) were not consistently available. Due to the small number of studies, we could not assess the source of heterogeneity via subgroup or meta-regression analysis. Lastly, most study populations were from high-income countries, so the findings may not be fully applicable to other healthcare settings.

The present findings highlight the importance of proactive cardiovascular risk assessments and strategies to prevent heart failure in patients with schizophrenia and bipolar disorder. Given the substantially increased risk, routine screening for cardiometabolic factors, such as blood pressure, blood glucose levels, lipid profiles, body mass index, and physical activity, should be incorporated into psychiatric care. Multidisciplinary teams, including psychiatrists, primary care physicians, cardiologists, and nursing personnel can facilitate earlier detection and management of cardiovascular conditions. Additionally, selecting and monitoring psychotropic medications carefully to minimize metabolic side effects is crucial. Recognizing severe mental illness as an independent cardiovascular risk factor may enable targeted prevention efforts and help lower the incidence of heart failure within this at-risk group. In inpatient and community settings alike, psychiatric and primary care nurses can facilitate early recognition of cardiovascular symptoms, coordinate multidisciplinary referrals, and provide patient-centered education tailored to the cognitive and psychosocial needs of individuals with schizophrenia and bipolar disorder.

## CONCLUSION

The current results demonstrate that both schizophrenia and bipolar disorder are independently associated with a significantly increased risk of incident heart failure. The findings remained robust in sensitivity analyses. However, the substantial heterogeneity noted in the analysis is a cause of concern and results must be interpreted with caution. Further robust cohort studies shall supplement the present evidence.
